# The Pathogenic Role of Low Range Repeats in SCA17

**DOI:** 10.1371/journal.pone.0135275

**Published:** 2015-08-12

**Authors:** Jung Hwan Shin, Hyeyoung Park, Gwan Hee Ehm, Woong Woo Lee, Ji Young Yun, Young Eun Kim, Jee-Young Lee, Han-Joon Kim, Jong-Min Kim, Beom Seok Jeon, Sung-Sup Park

**Affiliations:** 1 Department of Neurology, Seoul National University College of Medicine, Seoul National University Hospital, Seoul, Republic of Korea; 2 Department of Laboratory medicine, Seoul National University College of Medicine, Seoul National University Hospital, Seoul, Republic of Korea; 3 Department of Neurology, Seoul National University College of Medicine, Seoul National University Bundang Hospital, Seongnam, Republic of Korea; 4 Department of Neurology, Hallym University Sacred Heart Hospital, Hallym University College of Medicine, Anyang, Republic of Korea; 5 Department of Neurology, Myongi Hospital, Goyang, Republic of Korea; 6 Department of Neurology, Ewha Womans University School of Medicine and Ewha Medical Research Institute, Seoul, Republic of Korea; 7 Department of Neurology, Seoul National University Boramae Hospital, Seoul, South Korea; 8 Department of Neurology, Eulji General Hospital, Eulji University, Seoul, South Korea; University of Texas MD Anderson Cancer Center, UNITED STATES

## Abstract

**Introduction:**

SCA17 is an autosomal dominant cerebellar ataxia with expansion of the CAG/CAA trinucleotide repeats in the TATA-binding protein (TBP) gene. SCA17 can have various clinical presentations including parkinsonism, ataxia, chorea and dystonia. SCA17 is diagnosed by detecting the expanded CAG repeats in the TBP gene; however, in the literature, pathologic repeat numbers as low as 41 overlap with normal repeat numbers.

**Methods:**

The subjects in this study included patients with involuntary movement disorders such as cerebellar ataxia, parkinsonism, chorea and dystonia who visited Seoul National University Hospital between Jan. 2006 and Apr. 2014 and were screened for SCA17. Those who were diagnosed with other genetic diseases or nondegenerative diseases were excluded. DNA from healthy subjects who did not have a family history of parkinsonism, ataxia, psychiatric symptoms, chorea or dystonia served as the control. In total, 5242 chromosomes from 2099 patients and 522 normal controls were analyzed.

**Results:**

The total number of patients included in the analysis was 2099 (parkinsonism, 1706; ataxia, 345; chorea, 37; and dystonia, 11). In the normal control, up to 44 repeats were found. In the 44 repeat group, there were 7 (0.3%) patients and 1 (0.2%) normal control. In 43 repeat group, there were 8 (0.4%) patients and 2 (0.4%) normal controls. In the 42 repeat group, there were 16 (0.8%) patients and 3 (0.6%) normal controls. In 41 repeat group, there were 48 (2.3%) patients and 8 (1.5%) normal controls. Considering the overlaps and non-significant differences in allelic frequencies between the patients and the normal controls with low-expansions, we could not determine a definitive cutoff value for the pathologic CAG repeat number of SCA17.

**Conclusion:**

Because the statistical analysis between the normal controls and patients with low range expansions failed to show any differences so far, we must consider that clinical cases with low range expansions could be idiopathic movement disorders showing coincidental CAG/CAA expansions. Thus, we need to reconsider the pathologic role of low range expansions (41–42). Long term follow up and comprehensive investigations using autopsy and imaging studies in patients and controls with low range expansions are necessary to determine the cutoff value for the pathologic CAG repeat number of SCA17.

## Introduction

Spinocerebellar ataxia type 17 (SCA17) is an autosomal dominant cerebellar ataxia characterized by ataxia, psychiatric symptoms, parkinsonism and involuntary movement such as chorea and dystonia.[1] It is caused by an abnormal expansion of the CAG/CAA trinucleotide repeats in the TATA binding protein (TBP) gene located in chromosome 6.[2, 3]

Typical SCA17 presents with ataxia and cognitive decline.[3] However, some patients present with atypical symptoms such as a Huntington’s disease-like phenotype [4] and Parkinsonism.[5, 6] Even non ataxic features have been reported as well.[5, 7] It has been suggested that lower-ranging expansions of SCA17 are more likely to cause parkinsonism than ataxia.[8]

The cutoff value for the pathologic CAG repeat number of SCA17 has not been clearly elucidated.[9] Early reports proposed that SCA17 with a repeat number of 47 or more is a new disease entity.[3] The repeat number was then gradually lowered, and the currently accepted abnormal repeat number is 43 repeats or more.[9] However, some later studies suggested 42 repeats could be pathologic.[6, 10] Furthermore, there have been case reports of patients with even 41 repeats: one presenting with late onset progressive cerebellar ataxia [11]; one with late onset chorea and psychiatric symptoms;[12] and one with a rapidly progressing cognitive phenotype.[13] On the other hand, healthy controls with more than 42 repeats have been reported including 44 [6] and 45 repeats [7].

The majority of trinucleotide repeat disorders including Huntington’s disease [14] or other SCAs [15–17] have an intermediate zone with a repeat number below the cut off value for the pathologic repeat number. It is also called an allele with reduced penetrance. There are problems with the cut off values for several SCAs including SCA17.[14–16] In the case of Huntington’s disease, the pathologic CAG repeat number is known to be 40 or more, and 36–39 repeats are considered as alleles with reduced penetrance. Although expansions below 30 were considered normal [18], an autopsy confirmed case of Huntington’s disease with 29 repeats was reported [19]. Because the gap between normal and abnormal repeat numbers is very narrow in SCA17,[6] further investigation of the repeat numbers below the cutoff value for the pathologic CAG repeat number is necessary.

In the present study, we reviewed the SCA17 repeat numbers in our patients with movement disorders and compared the allele distribution with normal healthy controls to investigate cutoff value for the pathologic CAG repeat number of SCA17.

## Method

Retrospective analysis was done of patients with cerebellar ataxia, parkinsonism, chorea and dystonia who visited Seoul National University Hospital Movement Disorder Clinic from Jan. 2006 to Apr. 2014 and were tested for SCA17. Not all patients who visited our clinic were tested for SCA17. Parkinson disease (PD), multiple system atrophy (MSA), progressive supranuclear palsy (PSP), corticobasal syndrome (CBS) and dementia with Lewy bodies (DLB) were clinically diagnosed.[20–24 Those who had a parkinsonian feature but did not meet the diagnostic criteria of the above mentioned parkinsonian syndromes were classified as other parkinsonism.

All the patients were native Koreans. Blood samples were collected after written informed consent was obtained from each participant. The Institutional Review Board of Seoul National University Hospital approved this study. DNA from the healthy subjects who did not have a family history of parkinsonism, ataxia, chorea, dystonia or psychiatric features served as the control. In total, 5242 chromosomes from 2099 patients and 522 normal controls were analyzed.

### Molecular studies

Genomic DNA was extracted from peripheral blood leukocytes using a standard protocol. The allele size of SCA17 was determined as previously described.[6, 25, 26] Briefly, genomic DNA was extracted using a DNA isolation kit (Gentra PureGene; Gentra Systems Inc, Minneapolis, Minnesota). The allele sizes of SCA17 were determined by polymerase chain reaction (PCR) amplification and fragment analysis with the ABI PRISM 3100 Genetic Analyzer (Applied Biosystems, Foster City, California) and GeneMapper version 3.5 soft- ware.^13, 14^

PCR was performed with the following primers:

forward, 5’-ATGCCTTATGGCACTGGACTG-3’ (6-FAM labeled),

and reverse, 5’-CTGCTGGGACGTTGACTGCTG-3’.

To examine the interrupted sequences, the amplified fragment containing the CAG repeats was subcloned into the pCR2.1-TOPO vector (Invitrogen, Carlsbad, Cali- fornia) according to the manufacturer’s instructions. The PCR products from the genomic DNAs and more than 3 cloned fragments were sequenced bidirectionally on an ABI PRISM 3100 Genetic Analyzer with the BigDye Terminator Cycle Sequencing Ready Reaction Kit (version 3.1; Applied Biosystems).

### Statistical analysis

Independent t-test and Mann-Whitney test were used to compare variables between groups. Pearson Chi-square test and Fisher’s exact test were used to compare categorical variables. The level of statistical significance was set at p <0.05. Two sample Kolmogorov-Smirnov tests were used to compare the distributions of the cumulative age of onset between groups. The statistical package for the social sciences (SPSS 21.0) was used for all analyses.

## Results

The total number of patients included in the analysis was 2099 (classified by dominant clinical phenotype: parkinsonism, 1706; ataxia, 345; chorea, 37; and dystonia, 11). Parkinsonism consisted of PD (n = 1069, 50.9%), MSA-parkinsonian type (n = 153, 7.3%), PSP (n = 23, 1.1%), CBS (n = 6, 0.3%) and DLB (n = 6, 0.3%) and other Parkinsonism (n = 449, 21.4%). Ataxia consisted of MSA with cerebellar type (n = 125, 6.0%) and adult-onset cerebellar ataxia (n = 220, 10.5%).

The mean age of the normal controls (63.9 ± 9.1 years [standard deviation], range 38–87 years) was higher than that of the patients (61.3 ± 10.2 years [standard deviation], range 3–91 years) with statistical significance (p<0.0001).

**Fig 1:**
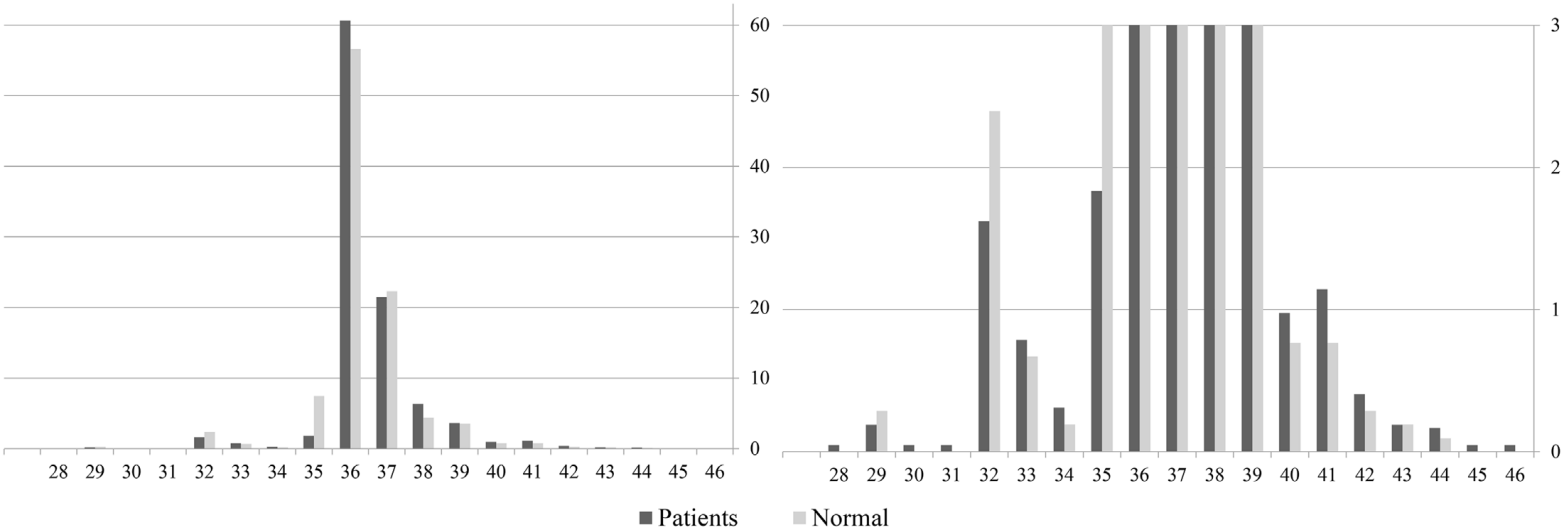
Distribution of CAG/CAA repeats in the TATA-binding protein gene in normal controls and patients. Patients are shown as dark colored bar and normal control as light colored bar. Y axis represents the percentage of each allele. Left panel shows the whole distribution, while right panel focused on repeats with low percentage by limiting the y axis to 3 percent.

The number of repeats in SCA17 alleles ranged from 29 to 44 in the normal controls and 23 to 46 in the patients (Fig 1). The mode was 36 in both the patient and control group. Because we compared the allelic distributions between the patients and normal controls by each repeat number, the allelic percentage was quite similar although slightly larger in the patient group with a repeat number above 41 without any statistical significance (Table 1). Patients with low expansions showed variable clinical features including parkinsonian syndromes, cerebellar ataxia and chorea. Distribution and clinical diagnoses are summarized in Table 1. Comparisons of the allelic frequencies between the normal controls and the patients are presented in Tables 2 and 3. The clinical presentations of the patients are presented in S2 Table.

**Table 1:** Distribution and clinical diagnoses of patients with SCA17.

No. of repeats	Patient		Control	
	Phenotypes	%	phenotypes	%
46	2 (1 MSA, 1 Pism)	**0.1**	0	**0**
45	2 (2 PD)	**0.1**	0	**0**
44	7 (2 PD, 1 MSA, 2 Pism, 2 CA)	**0.3**	1	**0.2**
43	8 (4 PD, 3 Pism, 1 MSA)	**0.4**	2	**0.4**
42	16 (7 PD, 3 MSA, 2 HDL, 1 PSP, 2 Pism, 1 CA)	**0.8**	3	**0.6**
41	48 (27 PD, 9 Pism, 7 MSA, 3CA, 1 HDL, 1 CBS)	**2.3**	8	**1.5**
Cumulation/Total	83/2099		14/522	

Distributions are overlapped between the normal controls and patients. MSA: Multiple system atrophy, PD: Parkinson disease, CA: Cerebellar ataxia, Pism: Other parkinsonism, HDL: Huntington disease like symptom, CBS: Corticobasal syndrome

**Table 2:** Comparison of allelic frequencies between the normal controls and patients for each allele.

Allele	Allelic frequencies of patients.	Patients(%)	Allelic frequencies of normal controls.	Normal(%)	P value
46	2	0.05	0	0	1.000
45	2	0.05	0	0	1.000
44	7	0.2	1	0.1	1.000
43	8	0.2	2	0.2	1.000
42	17*	0.4	3	0.3	1.000
41	48	1.1	8	0.8	0.289
40	41	0.98	8	0.8	0.527
Total	4198	100	1044	100	

*There were 16 patients with 42 repeats, but TNR of the patient with 46 repeats(patient No.2 in S2 Table) was 46/42. So allelic frequency was counted as 17 in 42 repeats.

No.: Number of patients or controls.

**Table 3:** Comparison of allelic frequencies between the normal controls and patients by disease subtypes for each allele.

Allele	PD No (%)	MSA No (%)	Chorea No (%)	Other parkinsonism No (%)	Cerebellar ataxia No (%)	Normal No (%)
46	0 (0)	1 (0.2)	0 (0)	1 (0.1)	0 (0)	0 (0)
45	2 (0.1)	0 (0)	0 (0)	0 (0)	0 (0)	0 (0)
44	2 (0.1)	1 (0.2)	0 (0)	2 (0.2)	2 (0.5)	1 (0.1)
43	4 (0.2)	1 (0.2)	0 (0)	3 (0.3)	0 (0)	2 (0.2)
42	7 (0.3)	3 (0.5)	2 (2.7)*	3 (0.3)	1 (0.2)	3 (0.3)
41	27 (1.3)	7 (1.3)	1 (1.4)	9 (1.0)	3 (0.7)	8 (0.7)
Total	2138	556	74	898	440	1044

Allelic frequency of chorea with 42 repeats was significantly higher than that of the normal controls(*p = 0.033, analyzed with Fisher’s exact test)

No.: Number of patients or controls.

**Fig 2:**
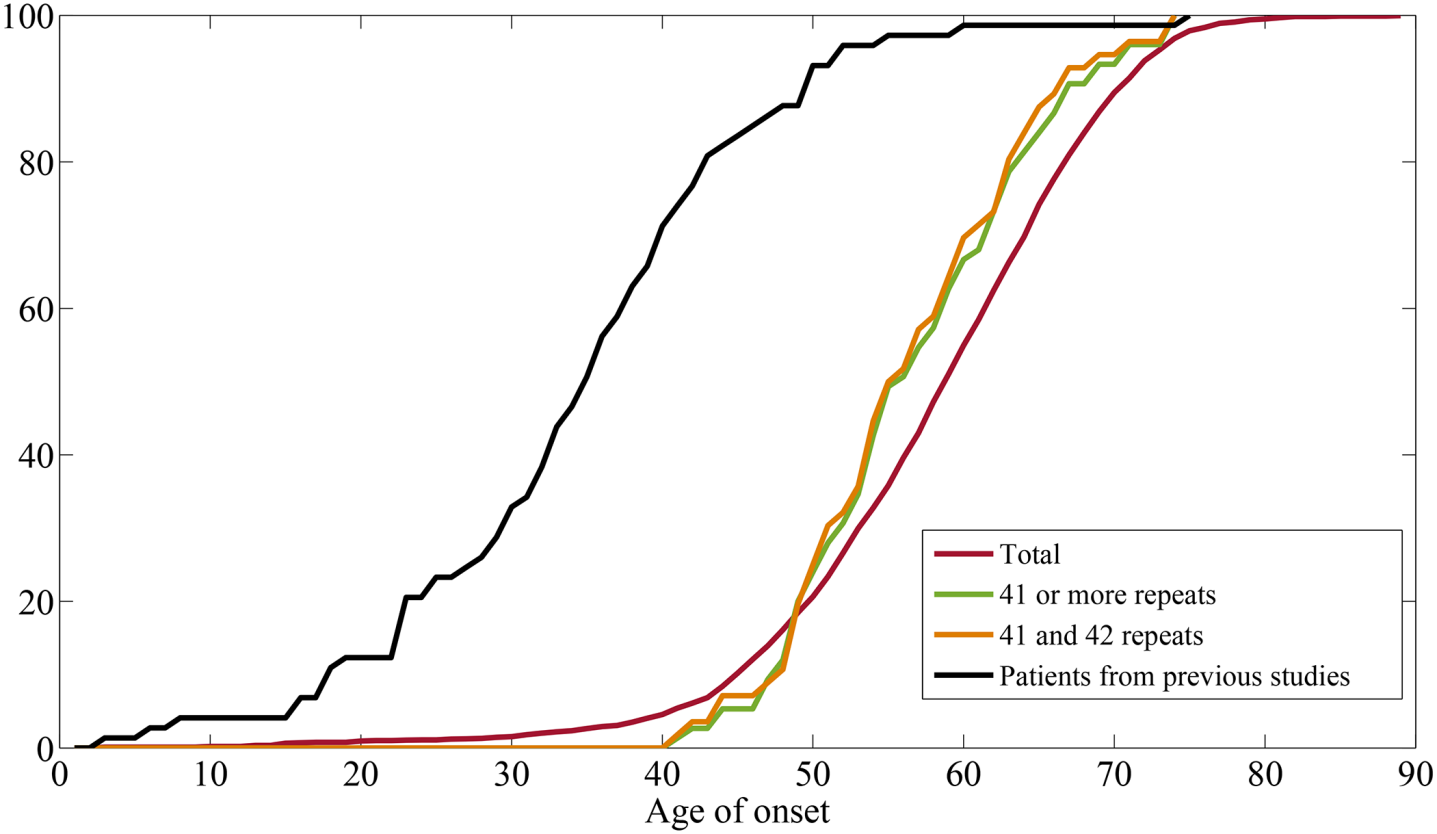
Distribution of the cumulative age of onset in patients. Forty-one or more repeats as well as 41 and 42 repeats showed a left shifted cumulative distribution compared to the total population, which was statistically significant by two-sample Kolmogorov-Smirnov test (p <0.001). The black line shows the patient populations collected from previous studies showing an earlier onset age compared to the total population from this study. (p<0.001)

Mean age of onset in the total patient population was 58.3 ± 10.7 years [standard deviation] and for patients with 41 or more repeats 57.1 ± 7.86 years [standard deviation] which were not statistically different. However, when plotting them with the cumulative age of onset, the distribution of patients with 41 or more repeats were shifted to left when compared to the total patient population which implies a relatively early onset age (p < 0.001). This was consistent when compared with only 41 and 42 repeats. (Fig 2).

We also analyzed the distribution of the age-of-onset included in previous literature that reported on SCA 17 and clearly stated the age of onset. Studies reporting 41 and 42 repeats were included as well. Eighty patients from 20 previous studies were included in the analysis. The mean repeat number was 48.2 ± 5.0 repeats [standard deviation]. Repeat numbers ranged from 41 to 66, and the onset age ranged from 3 to 75 years (details are summarized in S3 Table).

### 46 repeats

There were 2 patients with 46 repeats in our population. One was classified as MSA, and the other was diagnosed as other parkinsonism as described in the methods. They both showed cerebellar signs and parkinsonism but no psychiatric symptoms, cognitive impairment or chorea. They denied having a family history. There was atrophy in middle cerebellar peduncle and brainstem in MSA patient. The patient with other parkinsonism had a disease duration of 6 years without autonomic symptoms. DAT scan was not done in these patients (Patients 1 and 2 in S2 Table).

### 45 repeats

Two patients with 45 repeats were both diagnosed with Parkinson disease. There were no cerebellar signs as well as psychiatric symptoms, cognitive impairment or chorea. Both showed asymmetric onset of parkinsonian symptoms with good dopa responsiveness, which are typical features of idiopathic Parkinson disease. Age of onset was 67 years in one patient with a disease duration of 1 year; the other patient had a disease duration of 10 years with an age of onset of 45 years (Patients 3 and 4 in S2 Table).

### 44 repeats

In our normal population, the largest expansion was a 44 repeat. Even though asymptomatic, this 51 year old normal control showed a severe reduction in DAT binding, as previously reported by our group [6] There were 7 (0.3%) patients who showed parkinsonism as a dominant phenotype except for 2 cerebellar ataxic phenotypes (Table 1). Patient number 9 (S2 Table) with pure cerebellar ataxia showed a decreased striatal uptake in the FP-CIT PET, although she had no parkinsonism.

### 43 repeats

Two (0.4%) normal controls and 8 (0.4%) patients had 43 or more repeats which is currently accepted as the lower pathologic margin of SCA17. Comparing the allelic frequency between these two groups, there was no statistical significance. One of two normal controls showed a modest reduction in DAT binding also, reported by our group.[6] The other normal control was not tested for DAT binding.

### 42 repeats

Three (0.6%) normal controls and 16 (0.8%) patients had 42 repeats. The allelic percentage of the normal controls was 0.3% and 0.4% for the patients. In each disease subgroup, the allelic frequency of the PD patients was 0.3%, 0.5% for MSA and 2.7% for chorea (Table 3). The allelic frequency of chorea with 42 repeats was higher than that of the normal population with statistical significance (p = 0.033). The other correlation analyses showed no statistical difference between the groups.

One normal control showed a mild reduction in DAT binding considering his age,[6] two other normal controls were not tested for DAT binding. Patient number 23 showed marked cerebellar ataxia with cerebellar atrophy and familial history (3 of her 4 sisters were reported to show cerebellar ataxia). SCA 1, 2, 3, 6, 7, DRPLA and Friedreich’s ataxia were excluded in this patient. But unfortunately, genetic tests were not done in her sisters.

### 41 repeats

Eight (1.5%) normal controls and 48 (2.3%) patients had 41 repeats. The allelic percentage was 0.7% in the normal controls and 1.1% in the patients, which showed no statistical difference. Among the patients with 41 repeats, Patient number 63 showed parkinsonism with generalized chorea which appeared before medication. Huntington’s disease and DRPLA were excluded by the gene tests of this patient. There were no other identifiable secondary causes or abnormalities in the brain MRI. Patients 56 and 75 who also have 41 repeats, showed cerebellar ataxia with cerebellar and middle cerebellar peduncle atrophy in the brain MRI. The father of Patient 75 was reported to have cerebellar ataxia. Unfortunately the father was not tested for SCA17 because he passed away before the patient first visited the clinic.

When we compared the normal controls who were more than 55 years of age (N = 434) with the patients, the allelic frequencies and distributions were not different between the patients and the normal controls, similar to the above results.

Sequence analysis of the structure of the CAA/CAG repeats was done in 36 patients (10 in Kim et al [6] and 8 in Yun et al [8]), as well as in 18 additional patients with ataxia, parkinsonism or chorea (listed in S1 Table), which all showed an interrupted sequence except for 2 patients included in Yun et al [8].

## Discussion

The objective of this study was to investigate the cutoff value for the pathologic CAG repeat number of SCA17 and compare the distribution of the CAG/CAA repeats between patients and normal controls.

The normal controls were older than the patient group in our population (63.9± 9.1 versus 61.3 ± 10.2). The age of onset for SCA17 is known to range from age 3 to 75 years from a previous review.[27] Hence, normal controls with a young age can be presymptomatic patients. Minimizing the chance of including presymptomatic patients in the normal control group seems to be acceptable. In addition, we did a separate analysis for the normal controls aged over 55 years to minimize the problem of presymptomatic cases, which gave the same results.

In our population, 83 (4.0%) among 2099 patients showed repeats from 41 to 46. Among the 83 patients, 64 (3.0%) had 41 or 42 repeats which is considered controversial.

Although we found interesting cases harboring 41 or 42 repeats, the allelic frequencies for repeats as high as 43 were similar between the patients and controls. Additionally, the normal controls showed expansions as large as 44. Thus, we could not determine a definitive cutoff value for the pathologic CAG repeat number of SCA17. A recent report [28] compared the allelic distribution of normal controls with autosomal dominant PD in polyglutamine disease that included SCA17. They found a significantly different distribution in SCA 2 but not in the other genes. Their result is consistent with our results for SCA17.

Comparing the age of onset within a patient group, patients with low copy repeats showed an earlier onset age in the cumulative distribution. From populations collected from previous studies, it seems clear that the cumulative distributions show an early onset age in patients with 41 or more repeats from previous studies. This is consistent with previous studies showing a negative correlation between the repeat number and onset age.[29]

This may imply that low copy repeats play a role as susceptible factors in disease thus lowering the age of onset; however, considering the relatively small number of patients with 41 or more repeats (n = 83) compared to the total population (n = 2099), cautious interpretation is needed.

Current evidence, which suggests a pathologic role for SCA17 with low expansions, is mostly based on case descriptions showing movement disorders or psychiatric symptoms accompanied by CAG expansions in TBP.[10–13] Because the statistical analysis between the normal controls and patients with low expansion repeats failed to show any differences so far, we must consider that clinical cases with low expansion repeats could be idiopathic movement disorders showing coincidental CAG/CAA expansions. Thus, we need to reconsider the pathologic role of low-expansions(41–42).

To establish a definite cut off value for a pathologic CAG repeat number, the following evidence must be considered. Repeat numbers which have never been reported in a normal population or repeats with pathologic confirmation of cerebral tissue or repeats showing different allelic distributions compared with normal controls might be considered as cut offs. The largest expansion reported in a normal population is 45.[7] Additionally, because there is a lack of pathological and statistical evidence suggesting a cut off value for low expansions of SCA17 so far, a definite cut off value can be set as 46 or more. Forty-one through 45 should be considered as the intermediate range and needs cautious interpretation.

To further understand the cutoff value for the pathologic CAG repeat number of SCA17, autopsy results from deceased patients and normal controls with low expansion repeats should be investigated. Whether patients or asymptomatic normal controls with low-range expansions have intra-nuclear polyglutamine inclusions might be the key finding to solving this problem. There could be a possibility of somatic mosaicism where the gene structure such as repeat numbers can be distinct in the central nervous system and peripheral tissue. There have been some cases including a care report about somatic mosaicism in Parkinson disease [30, 31] Autopsy studies with extensive genetic testing will be necessary. Additionally, functional imaging such as dopamine transporter imaging for low expansion repeats including 41 in normal controls with serial follow up might reveal a subclinical pathology and provide us with more evidence for a clear cutoff value for the pathologic CAG repeat number of SCA17.

The limitation of our study includes the possibility that statistical analysis may have been underpowered by small number of patients in each repeats. Also, there could be a possibility of co-segregation of the patients in the familial pedigree with similar presentation. However, the number of patients with low copy repeats and a positive family history in our study was too small to consider co-segregation analysis.

Our results show that the pathologic CAG repeat number for SCA17 is not clear, and we should carefully examine the pathologic role of low-expansions in SCA17. Long term follow up observation and comprehensive investigations using autopsy and imaging studies in patients and controls with low expansion repeats are necessary to elucidate a definitive cut-off value for the pathologic CAG repeat number of SCA17.

## Supporting Information

S1 Table Sequence analysis of the CAA/CAG repeats in 18 patients. Those who were tested all had the interrupted form of the repeat.
(DOCX)

S2 Table Clinical characteristics of the patients with 41 or more repeats of SCA17. NI: no information, ND: Not done, WNL: within normal limit, SVD: small vessel disease.
(DOCX)

S3 Table Clinical characteristics of patients from previous studies with 41 or more repeats of SCA17. PD: Parkinson’s disease; HDL: Huntington’s disease-like phenotype.
(DOCX)
