# Supplementary material for: The Pathogenic Role of Low Range Repeats in SCA17
Source: PLoS One. 2015 Aug 12;10(8):e0135275. doi: 10.1371/journal.pone.0135275 (PMC4534202; doi:10.1371/journal.pone.0135275)
Supplement: S1 Table — Those who were tested all had the interrupted form of the repeat. (DOCX) [file pone.0135275.s001.docx]

Supplementary table 1

Sequence analyis of CAA/CAG repeats in 18 patients. Those who were tested were all interrupted form.

Supplementary table 1

| Patient  No. | Sex/Age | Diagnosis | TNR |  |
| --- | --- | --- | --- | --- |
| 3 | M/68 | PD | 45/36 | (CAG)3(CAA)3(CAG)9CAACAGCAA(CAG)25CAACAG |
|  |  |  |  | (CAG)3(CAA)3(CAG)6CAACAGCAA(CAG)19CAACAG |
| 8 | M/58 | Pism | 44/37 | (CAG)3(CAA)3(CAG)9CAACAGCAA(CAG)24CAACAG |
|  |  |  |  | (CAG)3(CAA)3(CAG)9CAACAGCAA(CAG)17CAACAG |
| 9 | F/64 | CA | 44/36 | (CAG)3(CAA)3(CAG)9CAACAGCAA(CAG)24CAACAG |
|  |  |  |  | (CAG)3(CAA)3(CAG)9CAACAGCAA(CAG)16CAACAG |
| 11 | F/48 | PD | 44/36 | (CAG)3(CAA)3(CAG)9CAACAGCAA(CAG)24CAACAG |
|  |  |  |  | (CAG)3(CAA)3(CAG)6CAACAGCAA(CAG)19CAACAG |
| 17 | F/68 | MSA | 43/36 | (CAG)3(CAA)3(CAG)9CAACAGCAA(CAG)23CAACAG |
|  |  |  |  | (CAG)3(CAA)3(CAG)9CAACAGCAA(CAG)16CAACAG |
| 18 | F/58 | PD | 43/37 | (CAG)3(CAA)3(CAG)9CAACAGCAA(CAG)23CAACAG |
|  |  |  |  | (CAG)3(CAA)3(CAG)8CAACAGCAA(CAG)18CAACAG |
| 20 | M/66 | MSA | 43/36 | (CAG)3(CAA)3(CAG)9CAACAGCAA(CAG)22CAACAG |
|  |  |  |  | (CAG)3(CAA)3(CAG)8CAACAGCAA(CAG)18CAACAG |
| 23 | F/64 | CA | 42/37 | (CAG)3(CAA)3(CAG)9CAACAGCAA(CAG)22CAACAG |
|  |  |  |  | (CAG)3(CAA)3(CAG)8CAACAGCAA(CAG)18CAACAG |
| 31 | M/66 | PD | 42/36 | (CAG)3(CAA)3(CAG)9CAACAGCAA(CAG)22CAACAG |
|  |  |  |  | (CAG)3(CAA)3(CAG)8CAACAGCAA(CAG)17CAACAG |
| 33 | M/76 | Chorea | 42/37 | (CAG)3(CAA)3(CAG)9CAACAGCAA(CAG)22CAACAG |
|  |  |  |  | (CAG)3(CAA)3(CAG)8CAACAGCAA(CAG)18CAACAG |
| 34 | F/59 | MSA | 42/37 | (CAG)3(CAA)3(CAG)9CAACAGCAA(CAG)22CAACAG |
|  |  |  |  | (CAG)3(CAA)3(CAG)6CAACAGCAA(CAG)20CAACAG |
| 36 | F/68 | chorea | 41/37 | (CAG)3(CAA)3(CAG)9CAACAGCAA(CAG)21CAACAG |
|  |  |  |  | (CAG)3(CAA)3(CAG)6CAACAGCAA(CAG)20CAACAG |
| 37 | M/51 | MSA | 41/38 | (CAG)3(CAA)3(CAG)9CAACAGCAA(CAG)21CAACAG |
|  |  |  |  | (CAG)3(CAA)3(CAG)8CAACAGCAA(CAG)17CAACAG |
| 38 | M/72 | Pism | 41/38 | (CAG)3(CAA)3(CAG)9CAACAGCAA(CAG)3CAA(CAG)17CAACAG |
|  |  |  |  | (CAG)3(CAA)3(CAG)9CAACAGCAA(CAG)18CAACAG |
| 44 | M/51 | MSA | 41/36 | (CAG)3(CAA)3(CAG)9CAACAGCAA(CAG)21CAACAG |
|  |  |  |  | (CAG)3(CAA)3(CAG)9CAACAGCAA(CAG)16CAACAG |
| 55 | F/55 | CA | 41/36 | (CAG)3(CAA)3(CAG)9CAACAGCAA(CAG)21CAACAG |
|  |  |  |  | (CAG)3(CAA)3(CAG)8CAACAGCAA(CAG)17CAACAG |
| 75 | M/59 | CA | 41/36 | (CAG)3(CAA)3(CAG)9CAACAGCAA(CAG)21CAACAG |
|  |  |  |  | (CAG)3(CAA)3(CAG)9CAACAGCAA(CAG)16CAACAG |
| 79 | M/68 | CA | 41/36 | (CAG)3(CAA)3(CAG)9CAACAGCAA(CAG)21CAACAG |
|  |  |  |  | (CAG)3(CAA)3(CAG)8CAACAGCAA(CAG)17CAACAG |
