# Supplementary material for: The Pathogenic Role of Low Range Repeats in SCA17
Source: PLoS One. 2015 Aug 12;10(8):e0135275. doi: 10.1371/journal.pone.0135275 (PMC4534202; doi:10.1371/journal.pone.0135275)
Supplement: S2 Table — NI: no information, ND: Not done, WNL: within normal limit, SVD: small vessel disease. (DOCX) [file pone.0135275.s002.docx]

Supplementary table 2.

Clinical characteristics of patients with 41 or more repeats of SCA 17. N.I : no information, ND : Not done, WNL : within normal limit, SVD : small vessel disease

Supplementary table 2.

| No | Sex/Age | TNR | Diagnosis | Cerebellar | Parkinsonism | Psychiatric | Cognitive impairment | Chorea | Brain MRI | DAT/SPECT | Family History |
| --- | --- | --- | --- | --- | --- | --- | --- | --- | --- | --- | --- |
| 1 | F/57 | 46/36 | MSA | (+) | (+) | (-) | (-) | (-) | MCP atrophy | ND | (-) |
| 2 | F/61 | 46/42 | Pism | (+) | (+) | (-) | (-) | (-) | WNL | ND | (-) |
| 3 | M/68 | 45/36 | PD | (-) | (+) | (-) | (-) | (-) | ND | ND | N.I |
| 4 | F/55 | 45/37 | PD | (-) | (+) | (-) | (-) | (-) | WNL | ND | (-) |
| 5 | F/60 | 44/36 | CA | (+) | (-) | (-) | N.I | (-) | MCP,cerebellar atrophy | Decreased Striatal uptake | (-) |
| 6 | M/66 | 44/36 | Pism | (+) | (+) | (-) | (-) | (-) | SVD | ND | (-) |
| 7 | M/71 | 44/36 | PD | (-) | (+) | (-) | (-) | (-) | WNL | ND | (-) |
| 8 | M/58 | 44/37 | Pism | (-) | (+) | (-) | N.I | (-) | SVD | Decreased Striatal uptake | (-) |
| 9 | F/64 | 44/36 | CA | (+) | (-) | (-) | (-) | (-) | Cerebellar atrophy | Decreased Striatal uptake | (-) |
| 10 | M/74 | 44/36 | MSA | (+) | (-) | (-) | (-) | (-) | SVD | ND | (-) |
| 11 | F/48 | 44/36 | PD | (-) | (+) | (-) | (-) | (-) | ND | ND | (-) |
| 12 | M/43 | 43/36 | PD | (-) | (+) | (-) | (-) | (-) | ND | ND | (-) |
| 13 | F/68 | 43/36 | PD | (-) | (+) | (+) | N.I | (-) | putaminal iron deposition | ND | (-) |
| 14 | M/80 | 43/36 | Pism | (-) | (+) | (+) | (+) | (-) | SVD | ND | (-) |
| 15 | F/72 | 43/36 | Pism | (+) | (+) | (-) | (-) | (-) | SVD | ND | (-) |
| 16 | F/55 | 43/35 | PD | (-) | (+) | (-) | (-) | (-) | WNL | Decreased Striatal uptake | (-) |
| 17 | M/60 | 43/36 | MSA | (-) | (+) | (-) | (+) | (-) | MCP,cerebellar atrophy | ND | (-) |
| 18 | F/58 | 43/37 | PD | (-) | (+) | (-) | (-) | (-) | ND | ND | (-) |
| 19 | F/50 | 43/36 | Pism | (-) | (+) | (-) | (-) | (-) | ND | ND | (-) |
| 20 | M/66 | 42/37 | MSA | (+) | (-) | (-) | (-) | (-) | MCP,cerebellar atrophy | ND | (-) |
| 21 | M/61 | 42/36 | PD | (-) | (+) | (-) | (-) | (-) | ND | ND | (-) |
| 22 | M/84 | 42/37 | Pism | (-) | (+) | (-) | (+) | (-) | putaminal iron deposition | ND | N.I |
| 23 | F/64 | 42/37 | CA | (+) | (-) | (-) | N.I | (-) | Cerebellar atrophy | ND | (+) |
| 24 | M/77 | 42/36 | Pism | (-) | (+) | (-) | N.I | (-) | SVD | ND | N.I |
| 25 | F/58 | 42/36 | PD | (-) | (+) | (-) | (-) | (-) | WNL | ND | (-) |
| 26 | M/70 | 42/36 | PSP | (+) | (-) | (-) | N.I | (-) | WNL | ND | (-) |
| 27 | M/72 | 42/38 | PD | (-) | (+) | (-) | (-) | (-) | SVD | ND | (-) |
| 28 | M/85 | 42/36 | chorea | (-) | (-) | (+) | (+) | (+) | WNL | ND | N.I |
| 29 | M/59 | 42/36 | PD | (-) | (+) | (-) | (-) | (-) | SVD | Decreased Striatal uptake | (-) |
| 30 | M/66 | 42/36 | MSA | (-) | (+) | (-) | (-) | (-) | Cerebellar atrophy | WNL | (-) |
| 31 | M/66 | 42/36 | PD | (-) | (+) | (+) | (-) | (-) | SVD | Decreased Striatal uptake | (-) |
| 32 | F/53 | 42/40 | PD | (-) | (+) | (+) | N.I | (-) | WNL | Decreased Striatal uptake | (+) |
| 33 | M/76 | 42/37 | Chorea | (-) | (-) | (-) | (-) | (+) | SVD | ND | (+) |
| 34 | F/68 | 42/37 | MSA | (-) | (+) | (-) | N.I | (-) | SVD | ND | (-) |
| 35 | F/56 | 42/38 | PD | (-) | (+) | (-) | (-) | (-) | WNL | ND | N.I |
| 36 | F/69 | 41/37 | chorea | (-) | (-) | (+) | (-) | (+) | Right frontal cerebromalacia | ND | N.I |
| 37 | M/52 | 41/38 | MSA | (+) | (+) | (+) | (-) | (-) | WNL | ND | N.I |
| 38 | M/73 | 41/38 | Pism | (-) | (+) | (-) | (-) | (-) | ND | Decreased Striatal uptake | N.I |
| 39 | F/77 | 41/37 | PD | (-) | (+) | (-) | (-) | (-) | SVD | ND | N.I |
| 40 | M/63 | 41/37 | PD | (-) | (+) | (-) | (-) | (-) | SVD | ND | N.I |
| 41 | M/61 | 41/36 | Pism | (-) | (+) | (+) | (-) | (-) | SVD | ND | N.I |
| 42 | F/75 | 41/40 | PD | (-) | (+) | (+) | (-) | (-) | SVD, old infarction in PICA territory | ND | (-) |
| 43 | F/59 | 41/36 | PD | (-) | (+) | (+) | (-) | (-) | SVD | ND | (-) |
| 44 | F/61 | 41/36 | MSA | (+) | (-) | (-) | (-) | (-) | cerebellar atrophy | ND | (-) |
| 45 | F/60 | 41/38 | PD | (-) | (+) | (-) | (-) | (-) | SVD | ND | (-) |
| 46 | F/69 | 41/36 | PD | (-) | (+) | (+) | (-) | (-) | WNL | ND | (-) |
| 47 | M/71 | 41/36 | PD | (-) | (+) | (+) | (-) | (-) | WNL | ND | (-) |
| 48 | M/61 | 41/36 | MSA | (-) | (+) | (-) | (-) | (-) | WNL | ND | N.I |
| 49 | M/61 | 41/36 | Pism | (-) | (+) | (-) | (-) | (-) | SVD | ND | (-) |
| 50 | F/67 | 41/36 | PD | (-) | (+) | (-) | (-) | (-) | ND | ND | (-) |
| 51 | F/76 | 41/37 | PD | (-) | (+) | (-) | (-) | (-) | ND | ND | (-) |
| 52 | M/53 | 41/37 | Pism | (-) | (+) | (+) | (-) | (-) | WNL | ND | N.I |
| 53 | F/74 | 41/36 | PD | (-) | (+) | (-) | (-) | (-) | ND | ND | (-) |
| 54 | F/64 | 41/39 | Pism | (+) | (+) | (-) | (-) | (-) | SVD | ND | (-) |
| 55 | F/57 | 41/36 | CA | (+) | (-) | (-) | (-) | (-) | SVD | ND | N.I |
| 56 | F/54 | 41/36 | CA | (+) | (-) | (-) | (-) | (-) | MCP,cerebellar atrophy | ND | N.I |
| 57 | F/76 | 41/36 | PD | (-) | (+) | (+) | (+) | (-) | WNL | ND | (-) |
| 58 | M/54 | 41/35 | Pism | (-) | (+) | (-) | (-) | (-) | SVD | ND | (-) |
| 59 | M/54 | 41/36 | MSA | (+) | (-) | (-) | (-) | (-) | cerebellar atrophy | ND | (-) |
| 60 | F/56 | 41/36 | PD | (-) | (+) | (-) | (-) | (-) | SVD | ND | (-) |
| 61 | M/65 | 41/36 | MSA | (+) | (+) | (-) | (-) | (-) | WNL | ND | (-) |
| 62 | F/53 | 41/37 | PD | (-) | (+) | (-) | (-) | (-) | WNL | ND | (-) |
| 63 | M/65 | 41/32 | Pism | (-) | (+) | (-) | (-) | (+) | SVD | ND | (+) |
| 64 | M/59 | 41/36 | Pism | (-) | (+) | (+) | (+) | (-) | putaminal iron deposition | ND | (-) |
| 65 | F/64 | 41/36 | PD | (-) | (+) | (-) | (-) | (-) | WNL | ND | (-) |
| 66 | F/58 | 41/36 | R/O CBS | (-) | (+) | (+) | (+) | (-) | WNL | ND | (-) |
| 67 | M/73 | 41/36 | MSA | (-) | (+) | (+) | (-) | (-) | SVD | Decreased Striatal uptake | (+) |
| 68 | F/67 | 41/36 | PD | (-) | (+) | (-) | (+) | (-) | ND | ND | (-) |
| 69 | M/74 | 41/37 | PD | (-) | (+) | (+) | (-) | (-) | SVD | Decreased Striatal uptake | (-) |
| 70 | M/66 | 41/36 | PD | (-) | (+) | (-) | (-) | (-) | SVD | ND | (-) |
| 71 | F/71 | 41/36 | MSA | (+) | (+) | (-) | (-) | (-) | diffuse brain atrophy | ND | (-) |
| 72 | F/61 | 41/38 | PD | (-) | (+) | (+) | (-) | (-) | WNL | ND | (-) |
| 73 | M/68 | 41/38 | PD | (-) | (+) | (-) | (-) | (-) | ND | ND | (+) |
| 74 | M/47 | 41/38 | PD | (-) | (+) | (-) | (-) | (-) | SVD | ND | (-) |
| 75 | M/60 | 41/36 | CA | (+) | (-) | (-) | (-) | (-) | MCP,cerebellar atrophy | ND | (+) |
| 76 | M/51 | 41/37 | PD | (-) | (+) | (-) | (-) | (-) | ND | Decreased Striatal uptake | (-) |
| 77 | M/45 | 41/36 | PD | (-) | (+) | (-) | (-) | (-) | WNL | nd | (-) |
| 78 | M/73 | 41/37 | PD | (-) | (+) | (-) | (-) | (-) | WNL | nd | (-) |
| 79 | M/69 | 41/36 | CA | (+) | (+) | (-) | (-) | (+) | WNL | WNL | (-) |
| 80 | F/55 | 41/36 | PD | (-) | (+) | (+) | (-) | (-) | ND | ND | (-) |
| 81 | M/69 | 41/36 | PD | (-) | (+) | (+) | (-) | (-) | ND | ND | (-) |
| 82 | M/55 | 41/37 | PD | (-) | (+) | (-) | (-) | (-) | ND | ND | (-) |
| 83 | M/49 | 41/37 | PD | (-) | (+) | (-) | (-) | (-) | WNL | Decreased Striatal uptake | (-) |
