# Supplementary material for: The Pathogenic Role of Low Range Repeats in SCA17
Source: PLoS One. 2015 Aug 12;10(8):e0135275. doi: 10.1371/journal.pone.0135275 (PMC4534202; doi:10.1371/journal.pone.0135275)
Supplement: S3 Table — PD: Parkinson’s disease; HDL: Huntington’s disease-like phenotype. (DOCX) [file pone.0135275.s003.docx]

Supplementary table 3.

| No | Sex | TNR | Diagnosis/  Presenting symptom | Cere  -bellar | Parkinson  -ism | Psychiatric | Cognitive impairment | Chorea | Age  of onset |
| --- | --- | --- | --- | --- | --- | --- | --- | --- | --- |
| Bauer et al, 2004[^1^](#_ENREF_1) | M | 52/40 | Hyperkinesia | (-) | (-) | (-) | (-) | (-) | 50 |
|  | F | 51/35 | Ataxia | (+) | (-) | (+) | (+) | (-) | 32 |
|  | M | 50/36 | Ataxia | (+) | (-) | (-) | (+) | (-) | 32 |
|  | M | 48/37 | Ataxia | (+) | (-) | (-) | (+) | (-) | 35 |
|  | F | 46/36 | Ataxia | (+) | (-) | (-) | (+) | (+) | 36 |
|  | M | 46/35 | Ataxia | (+) | (-) | (+) | (-) | (+) | 37 |
|  | M | 46/35 | HDL | (+) | (-) | (-) | (+) | (+) | 38 |
| Brockmann et al, 2012[^2^](#_ENREF_2) | F | 54 | Ataxia | (+) | (-) | (-) | (-) | (-) | 36 |
|  | M | 51 | Ataxia | (+) | (-) | (-) | (-) | (-) | 40 |
|  | M | 45 | Ataxia | (+) | (-) | (-) | (-) | (-) | 46 |
|  | M | 54 | Ataxia | (+) | (-) | (-) | (-) | (-) | 33 |
|  | M | 51 | Ataxia | (+) | (-) | (-) | (-) | (-) | 35 |
| Silveira et al, 2002[^3^](#_ENREF_3) | M | 43 | Ataxia | (+) | (-) | (-) | (+) | (-) | 52 |
| Fujigasaki et al, 2001[^4^](#_ENREF_4) | M | 46 | Ataxia | (+) | (-) | (-) | (+) | (-) | 50 |
|  | M | 46 | Ataxia | (+) | (-) | (-) | (+) | (+) | 37 |
|  | F | 46/37 | Ataxia | (+) | (-) | (-) | (-) | (-) | 55 |
|  | F | 46/39 | Ataxia | (+) | (-) | (-) | (-) | (-) | 34 |
| Gunther et al, 2004[^5^](#_ENREF_5) | M | 50/37 | Ataxia | (+) | (-) | (-) | (-) | (-) | 31 |
| Koide et al,1999[^6^](#_ENREF_6) | F | 63/36 | Ataxia | (+) | (-) | (-) | (+) | (-) | 6 |
| Lin et al, 2007 | M | 45/39 | Ataxia | (+) | (+) | (-) | (-) | (-) | 43 |
| Nakamura et al, 2001[^7^](#_ENREF_7) | M | 55/37 | Ataxia | (+) | (+) | (-) | (+) | (-) | 19 |
|  | F | 55/39 | Ataxia | (+) | (+) | (-) | (+) | (-) | 25 |
|  | M | 48/36 | Ataxia | (+) | (+) | (-) | (+) | (-) | 39 |
|  | M | 48/36 | Ataxia | (+) | (+) | (-) | (+) | (+) | 48 |
|  | F | 47/36 | Ataxia | (+) | (+) | (-) | (+) | (+) | 40 |
|  | F | 47/32 | Ataxia | (+) | (+) | (-) | (+) | (-) | 28 |
| Oda et al, 2004[^8^](#_ENREF_8) | F | 56/36 | Ataxia | (+) | (-) | (-) | (+) | (-) | 30 |
|  | F | 48/36 | Ataxia | (+) | (-) | (-) | (+) | (-) | 27 |
|  | M | 47/44 | Ataxia | (+) | (-) | (-) | (+) | (-) | 25 |
|  | M | 47/36 | Ataxia | (+) | (-) | (-) | (+) | (-) | 42 |
|  | F | 45/37 | Ataxia | (+) | (-) | (-) | (-) | (-) | 52 |
|  | F | 45/36 | Ataxia | (+) | (+) | (-) | (+) | (+) | 60 |
|  | M | 44/36 | Ataxia | (+) | (+) | (-) | (+) | (+) | 29 |
|  | F | 43/33 | Ataxia | (+) | (-) | (-) | (-) | (-) | 47 |
| Rolfs et al, 2003[^9^](#_ENREF_9) | F | 49/37 | Parkinsonism | (-) | (+) | (+) | (-) | (-) | 38 |
|  | F | 49/36 | Ataxia | (+) | (-) | (+) | (+) | (-) | 16 |
|  | F | 49 | Ataxia | (+) | (-) | (+) | (+) | (-) | 16 |
|  | M | 54/37 | Ataxia | (+) | (-) | (+) | (+) | (+) | 36 |
|  | M | 54/37 | Ataxia | (+) | (-) | (-) | (+) | (-) | 18 |
|  | F | 54/37 | Ataxia | (+) | (-) | (+) | (-) | (+) | 23 |
|  | M | 54/34 | Ataxia | (+) | (-) | (-) | (-) | (-) | 18 |
|  | F | 45/39 | Ataxia | (+) | (+) | (+) | (-) | (-) | 30 |
|  | M | 45/37 | Ataxia | (+) | (-) | (-) | (-) | (-) | 43 |
|  | F | 45/37 | Ataxia | (+) | (-) | (-) | (-) | (+) | 30 |
|  | M | 45/36 | Ataxia | (+) | (-) | (-) | (-) | (-) | 23 |
|  | F | 54 | Ataxia | (+) | (-) | (-) | (+) | (-) | 33 |
|  | F | 54 | Ataxia | (+) | (-) | (-) | (+) | (-) | 33 |
|  | F | 54 | Ataxia | (+) | (-) | (+) | (-) | (+) | 8 |
|  | M | 54 | Ataxia | (+) | (-) | (-) | (-) | (-) | 18 |
| Salvatore et al, 2006[^10^](#_ENREF_10) | M | 53 | Ataxia | (+) | (+) | (+) | (+) | (+) | 23 |
|  | F | 51 | Ataxia | (+) | (+) | (+) | (+) | (+) | 40 |
|  | M | 52 | Ataxia | (+) | (+) | (+) | (+) | (+) | 40 |
| Toyoshima et al, 2004[^11^](#_ENREF_11) | M | 48/48 | Ataxia | (+) | (-) | (-) | (+) | (+) | 39 |
| Wu et al, 2004[^12^](#_ENREF_12) | F | 46 | PD | (-) | (+) | (-) | (-) | (+) | 75 |
| Zuhlke et al,2005[^13^](#_ENREF_13) | M | 53 | Ataxia | (+) | (-) | (-) | (-) | (+) | 41 |
|  | F | 52 | Ataxia | (+) | (-) | (-) | (-) | (+) | 50 |
| Zuhlke et al,2003[^14^](#_ENREF_14) | F | 48/38 | Ataxia | (+) | (-) | (-) | (+) | (+) | 38 |
|  | M | 48/38 | Ataxia | (+) | (-) | (-) | (+) | (-) | 43 |
|  | F | 48/37 | Ataxia | (+) | (-) | (-) | (+) | (-) | 36 |
|  | M | 48/37 | Ataxia | (+) | (-) | (-) | (+) | (+) | 33 |
| Stevanin et al, 2003[^15^](#_ENREF_15) | F | 46 | HDL | (+) | (+) | (-) | (+) | (+) | 23 |
|  | F | 44 | HDL | (+) | (-) | (-) | (+) | (+) | 29 |
| Maltecca et al, 2003[^16^](#_ENREF_16) | F | 53 | Ataxia | (+) | (-) | (+) | (+) | (-) | 35 |
|  | M | 53 | Ataxia | (+) | (-) | (+) | (+) | (-) | 34 |
|  | M | 53 | Ataxia | (+) | (-) | (+) | (+) | (-) | 23 |
|  | F | 66 | Ataxia | (+) | (-) | (+) | (+) | (-) | 3 |
| Alibardi et al, 2014[^17^](#_ENREF_17) | F | 41 | Chorea/  Psychiatric symptom. | (-) | (-) | (+) | (-) | (+) | 23 |
| Nanda et al, 2007[^18^](#_ENREF_18) | M | 41 | Ataxia | (+) | (-) | (+) | (+) | (-) | 50 |
| Nielsen et al, 2012[^19^](#_ENREF_19) | M | 41 | Dementia | (+) | (-) | (-) | (-) | (-) | 42 |
| Nolte et al, 2010[^20^](#_ENREF_20) | F | 42 | Ataxia | (+) | (-) | (-) | (-) | (-) | 41 |
|  | F | 42 | Ataxia | (+) | (-) | (-) | (+) | (-) | 32 |
|  | M | 42 | Ataxia | (+) | (-) | (-) | (+) | (-) | 45 |
|  | M | 42 | Ataxia | (+) | (-) | (-) | (-) | (-) | 44 |

1. Bauer P. Trinucleotide repeat expansion in SCA17/TBP in white patients with Huntington's disease-like phenotype. Journal of Medical Genetics 2004;41:230-232.

2. Brockmann K, Reimold M, Globas C, et al. PET and MRI reveal early evidence of neurodegeneration in spinocerebellar ataxia type 17. Journal of nuclear medicine : official publication, Society of Nuclear Medicine 2012;53:1074-1080.

3. Silveira I, Miranda C, Guimaraes L, et al. Trinucleotide repeats in 202 families with ataxia: a small expanded (CAG)n allele at the SCA17 locus. Archives of neurology 2002;59:623-629.

4. Fujigasaki H, Martin JJ, De Deyn PP, et al. CAG repeat expansion in the TATA box-binding protein gene causes autosomal dominant cerebellar ataxia. Brain : a journal of neurology 2001;124:1939-1947.

5. Gunther P, Storch A, Schwarz J, et al. Basal ganglia involvement of a patient with SCA 17--a new form of autosomal dominant spinocerebellar ataxia. Journal of neurology 2004;251:896-897.

6. Koide R, Kobayashi S, Shimohata T, et al. A neurological disease caused by an expanded CAG trinucleotide repeat in the TATA-binding protein gene: a new polyglutamine disease? Human molecular genetics 1999;8:2047-2053.

7. Nakamura K, Jeong SY, Uchihara T, et al. SCA17, a novel autosomal dominant cerebellar ataxia caused by an expanded polyglutamine in TATA-binding protein. Human molecular genetics 2001;10:1441-1448.

8. Oda M, Maruyama H, Komure O, et al. Possible reduced penetrance of expansion of 44 to 47 CAG/CAA repeats in the TATA-binding protein gene in spinocerebellar ataxia type 17. Archives of neurology 2004;61:209-212.

9. Rolfs A, Koeppen AH, Bauer I, et al. Clinical features and neuropathology of autosomal dominant spinocerebellar ataxia (SCA17). Annals of neurology 2003;54:367-375.

10. Salvatore E, Varrone A, Sansone V, et al. Characterization of nigrostriatal dysfunction in spinocerebellar ataxia 17. Movement disorders : official journal of the Movement Disorder Society 2006;21:872-875.

11. Toyoshima Y, Yamada M, Onodera O, et al. SCA17 homozygote showing Huntington's disease-like phenotype. Annals of neurology 2004;55:281-286.

12. Wu YR, Lin HY, Chen CM, et al. Genetic testing in spinocerebellar ataxia in Taiwan: expansions of trinucleotide repeats in SCA8 and SCA17 are associated with typical Parkinson's disease. Clinical genetics 2004;65:209-214.

13. Zuhlke C, Dalski A, Schwinger E, Finckh U. Spinocerebellar ataxia type 17: report of a family with reduced penetrance of an unstable Gln49 TBP allele, haplotype analysis supporting a founder effect for unstable alleles and comparative analysis of SCA17 genotypes. BMC medical genetics 2005;6:27.

14. Zuhlke C, Gehlken U, Hellenbroich Y, Schwinger E, Burk K. Phenotypical variability of expanded alleles in the TATA-binding protein gene. Reduced penetrance in SCA17? Journal of neurology 2003;250:161-163.

15. Stevanin G, Fujigasaki H, Lebre AS, et al. Huntington's disease-like phenotype due to trinucleotide repeat expansions in the TBP and JPH3 genes. Brain : a journal of neurology 2003;126:1599-1603.

16. Maltecca F, Filla A, Castaldo I, et al. Intergenerational instability and marked anticipation in SCA-17. Neurology 2003;61:1441-1443.

17. Alibardi A, Squitieri F, Fattapposta F, et al. Psychiatric onset and late chorea in a patient with 41 CAG repeats in the TATA-binding protein gene. Parkinsonism & related disorders 2014;20:678-679.

18. Nanda A, Jackson SA, Schwankhaus JD, Metzer WS. Case of spinocerebellar ataxia type 17 (SCA17) associated with only 41 repeats of the TATA-binding protein (TBP) gene. Movement disorders : official journal of the Movement Disorder Society 2007;22:436.

19. Nielsen TT, Mardosiene S, Lokkegaard A, et al. Severe and rapidly progressing cognitive phenotype in a SCA17-family with only marginally expanded CAG/CAA repeats in the TATA-box binding protein gene: a case report. BMC neurology 2012;12:73.

20. Nolte D, Sobanski E, Wissen A, Regula JU, Lichy C, Muller U. Spinocerebellar ataxia type 17 associated with an expansion of 42 glutamine residues in TATA-box binding protein gene. Journal of neurology, neurosurgery, and psychiatry 2010;81:1396-1399.
